# Myristicin from *Athamanta sicula* L.: A Potential Natural Antimicrobial Agent

**DOI:** 10.3390/antibiotics15010079

**Published:** 2026-01-13

**Authors:** Antonella Porrello, Alessia Sordillo, Natale Badalamenti, Giusy Castagliuolo, Giuseppe Bazan, Daniela Di Girolamo, Mario Varcamonti, Anna Zanfardino, Maurizio Bruno

**Affiliations:** 1 Department of Biological, Chemical and Pharmaceutical Sciences and Technologies (STEBICEF), Università degli Studi di Palermo, Viale delle Scienze, Ed. 17, 90128 Palermo, Italy; antonella.porrello@unipa.it (A.P.); natale.badalamenti@unipa.it (N.B.); giuseppe.bazan@unipa.it (G.B.); maurizio.bruno@unipa.it (M.B.); 2 Department of Biology, University of Naples Federico II, 80126 Naples, Italy; alessiasordillo2015@gmail.com (A.S.); giusy.castagliuolo@unina.it (G.C.); daniela.digirolamo@unina.it (D.D.G.); varcamon@unina.it (M.V.); 3 NBFC, National Biodiversity Future Center, 90133 Palermo, Italy

**Keywords:** antibiofilm activity, antimicrobial activity, Apiaceae, *Athamanta sicula* L., myristicin, *β*-Phellandrene

## Abstract

*Athamanta* L. is a small genus of the Apiaceae family, comprising only sixteen species and subspecies, which are distributed in the Canary Islands, Central Europe, and the Mediterranean basin. **Background/Objectives**: Since the time of Dioscurides, the species of this genus have been reported to have had several ethnopharmacological activities, and some of them are also used currently. *Athamanta sicula* L., growing in Italy, Tunisia, Algeria, and Morocco, is the only species of this genus present in Sicily. To further explore the phytochemical profile and biological properties of this species, the present study focused on the essential oil (EO) extracted from the aerial parts of wild *A. sicula* populations collected in central Sicily. **Methods**: The chemical composition of the EO, obtained by hydrodistillation, was determined by GC–MS analysis. The presence of myristicin was confirmed by isolation and by ^1^H-NMR spectroscopic characterization. **Results**: The EO and its main constituents have been tested for possible antimicrobial properties against several bacterial strains, showing MIC values in the of 15–30 mg/mL range, and the mechanism of action was further investigated, revealing membrane-targeting effects consistent with outer membrane permeabilization. In addition, antibiofilm activity (with up to ~80% inhibition of biofilm formation at sub-MICs), antioxidant potential (demonstrating dose-dependent radical scavenging activity), and biocompatibility with eukaryotic cells were assessed to provide a comprehensive pharmacological profile of *A. sicula* EO. Specifically, the most abundant constituent was myristicin (62.2%), the principal representative of the phenylpropanoid class (64.4%). Hydrocarbon monoterpenes represented the second class of the EO (27.4%), with *β*-phellandrene (12.2%) as the main compound. **Conclusions**: Myristicin emerged as the key contributor to the antimicrobial and antibiofilm activity of the EO. The obtained results highlight the relevance of *A. sicula* EO as a myristicin-rich essential oil with notable in vitro biological activity.

## 1. Introduction

The genus *Athamanta* L., belonging to the Apiaceae family, has complex nomenclatural history since several taxa assigned, in the past, to this genus have been later moved to other genera such as *Seseli* L., *Peucedanum* L., *Libanotis* Haller ex Zinn, *Schulzia* Spreng., *Cnidium* Cusson ex Juss., *Daucus* L., and *Cenolophium* W.D.J.Koch, just to mention the principal ones. *Athamanta* genus is distributed in Canary Islands, Central Europe to Mediterranean basin, and, according to POWO (Plants of the World Online), only sixteen species and subspecies have the rang of accepted taxa [1].

The pharmacological properties of *Athamanta* species have been reported since the time of Dioscurides, who mentions, in his book *De Materia Medica*, the use of the seeds and roots of *Athamanta cretensis* L. as antidotes and for the treatment of dermatologic, gastric, gynecologic, respiratory, and urologic problems [2]. At the present, it is utilized in the traditional Persian medicine in remedies associated with maternal well-being, uterine health, reproductive wellness, and anti-inflammatory [3]. The same species as well as *Athamanta macedonica* (L.) Spreng. is used in Europe against sclerosis and cancer [4].

**Figure 1:**
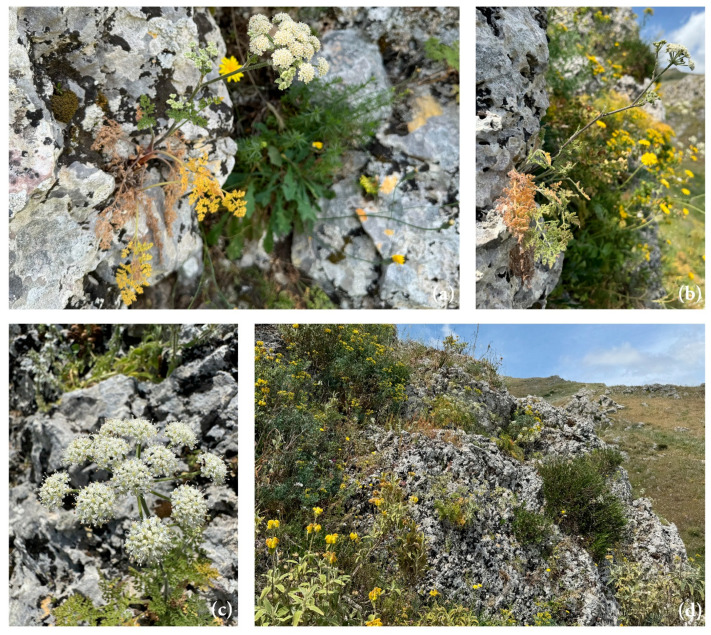
*Athamanta sicula*: (**a**) whole plant in its natural rocky habitat at the flowering stage; (**b**,**c**) compound umbel with fully developed white flowers; (**d**) characteristic rocky habitat of the species in Caltabellotta, Sicily, Italy. Photo by Prof. Giuseppe Bazan.

**Figure 2:**
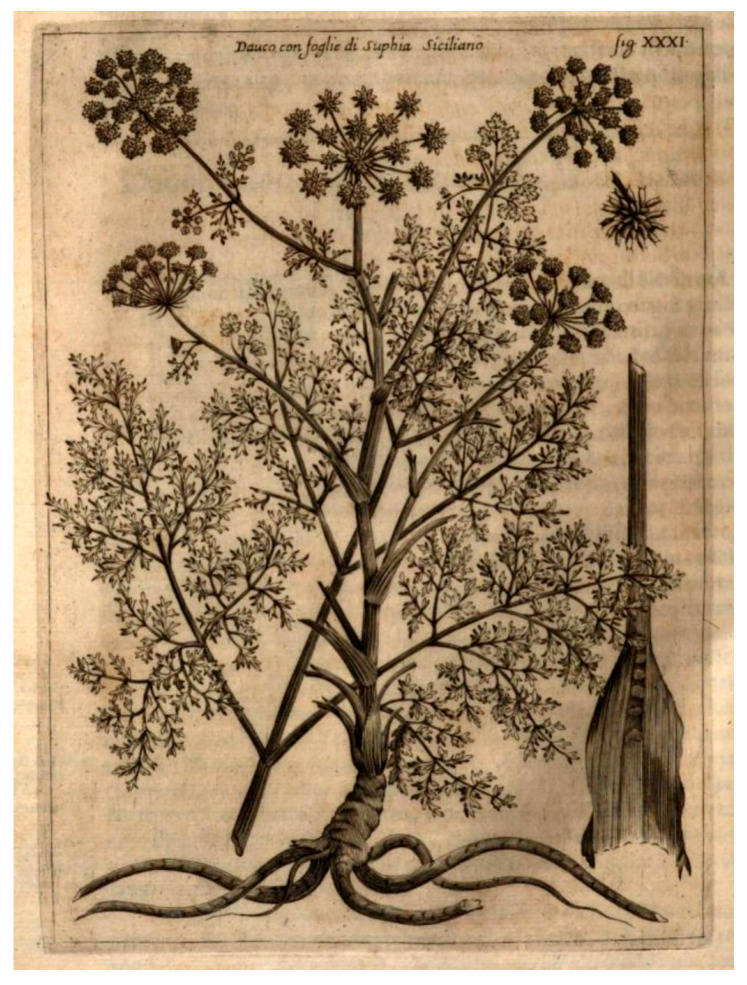
*Athamanta sicula*, illustrated as “Dauco con foglie di Sophia Siciliano”, in Giacomo Zanoni’s Istoria Botanica (1675), Tavola XXXI [7].

*Athamanta sicula* L. (Figure 1 and Figure 2) is the sole species of this genus growing in Sicily and, according to POWO [1], it is native to Sicily, the Italian Peninsula, Tunisia, Algeria, and Morocco, where it grows on calcareous cliffs and old walls, from sea level up to 1500 m of altitude [5,6].

Historical botanical treatises do not report any medicinal uses for this species, in contrast to other members of the genus *Athamanta*, such as *A. meum* L. (a synonym of *Meum athamanticum* Jacq.). According to Mattioli [8], the roots of this plant, when consumed either cooked in water or triturated in their raw state, were described and used to relieve renal and vesical obstructions (diuretic), to alleviate digestive disturbances (carminative), to reduce bodily pain, and to treat uterine disorders (emmenagogue).

However, in Sicilian folk medicine *Athamanta sicula* is used as a diuretic and to facilitate the elimination of kidney stones [9]. Indeed, the species is known in the Sicilian dialect as *spaccapetri* (stonebreaker), a name that may refer both to its ethnobotanical properties, related to the dissolution of renal calculi, and to its characteristic habitat, as the root grows within rock crevices. In Morocco, where it is known with the vernacular name *chkotat elhjar*, the poultice of the whole plant power is utilized against leprosy [10].

Only few papers have been published on the non-volatile metabolites of *Athamanta* taxa. They concern the fatty acid composition of fixed oils extract from the fruits of *Athamanta turbith* ssp. *hungarica* (Borbás) Tutin and *Athamanta turbith* ssp. *haynaldii* (Borbás & Uechtr.) Tutin [11], the isolation of several sesquiterpenes, phenylpropanoids, as well as sterols, triterpene, diterpene derivatives, and polyacetylenic compounds in the extracts of the aerial parts of *Athamunta vayredana* (Font Quer) C.Pardo (syn. *Seseli vayredanum* Font Quer) [12,13], and the identification of phthalides in roots of *Athamanta montana* (Webb ex Christ) Spalik & Wojew. & S.R.Downie (syn. *Todaroa montana* Webb ex Christ) [14].

On the other hands several investigations have been carried out into the essential oil (EO) compositions of some taxa [15,16,17,18,19,20,21,22,23], and they will be discussed later on.

The long-standing traditional use of *Athamanta* species for renal, dermatological, and gastrointestinal disorders provides a historical background that complements modern pharmacological research; however, contemporary investigations are increasingly driven by the urgent global challenge posed by antimicrobial resistance (AMR). The rapid emergence and dissemination of multidrug-resistant pathogens have rendered many conventional antibiotics less effective, prompting an intensified search for new antimicrobial agents with innovative mechanisms of action capable of overcoming resistance phenomena. In this context, the discovery of alternative molecular prototypes is recognized as a critical medical and scientific priority [24]

Natural products remain one of the most productive sources of chemically diverse bioactive compounds, offering structural complexity and functional versatility that are often absent in synthetic libraries [24]. In particular, plant-derived secondary metabolites have attracted renewed interest due to their broad-spectrum antimicrobial activity and their ability to act on multiple cellular targets [25]. Unlike conventional antibiotics, essential oil (EO) constituents frequently exert multitarget effects, including disruption of microbial membranes, inhibition of biofilm formation, modulation of quorum sensing, and attenuation of virulence factors, collectively reducing the selective pressure that drives resistance development [26].

A substantial body of evidence demonstrates that numerous EOs exhibit significant antimicrobial activity against pathogens associated with epithelial and intestinal infections, with minimum inhibitory concentration (MIC) values commonly reported in the low mg/mL range [19,25]. Moreover, Apiaceae-derived EOs have been shown to be particularly effective against uropathogenic microorganisms, including *Escherichia coli* and *Enterococcus* spp., which are among the principal causative agents of urinary tract and renal infections. In these cases, EO profiles rich in phenylpropanoids and monoterpenes have demonstrated consistent inhibitory effects, supporting their potential relevance in addressing infections historically linked to renal disorders [27,28].

Beyond ethnopharmacological considerations, these findings underscore the broader value of EOs as reservoirs of antimicrobial lead compounds with novel and complementary modes of action. Their capacity to interfere with microbial physiology at multiple levels positions plant-derived volatiles as promising candidates for the development of new anti-infective strategies aimed at mitigating AMR, either as standalone agents or in combination with existing antibiotics [24].

**Figure 3:**
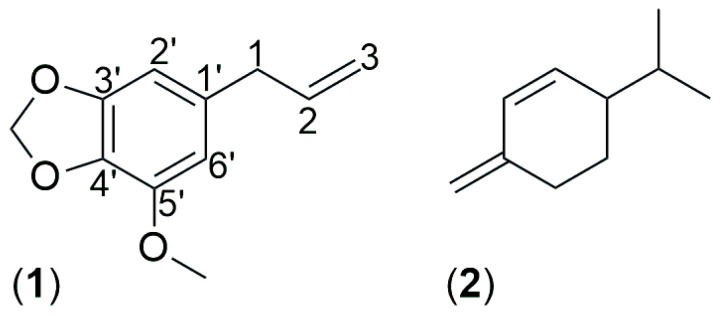
Chemical structures of myristicin (**1**) and *β*-phellandrene (**2**).

In order to expand the knowledge about the phytochemistry of *Athamanta* species, in this work was studied the chemical composition of *Athamanta sicula* aerial parts EO, collected at Caltabellotta, Sicily, Italy. Furthermore, was described the main biological properties of the EO and its major constituents, myristicin (**1**) and *β*-phellandrene (**2**) (Figure 3), were evaluated against a panel of bacterial strains representative of skin and intestinal microbiota, as well as on HaCaT human keratinocytes. This study was prompted by the traditional use of *Athamanta* species in the treatment of dermatological and gastrointestinal disorders since ancient times [2].

## 2. Results and Discussion

### 2.1. Chemical Composition of A. sicula EO

Hydrodistillation of the fresh aerial parts of *Athamanta sicula* gave a pale-yellow EO (**As**). Overall, twenty-nine compounds were identified in the sample, representing 98.1% of the total components. The components are listed in Table 1 according to their retention indices on a DB-5 MS column and they are classified based on their chemical structures into six classes.

**As** was mainly represented by phenylpropanoids (64.4%), with myristicin (62.2%) like the most abundant metabolites of the class and of the total composition. In the same class, the occurrence of low amount of apiole (2.2%) is worthy of mention. The second most abundant metabolite of the **As** was *β*-phellandrene (12.2%), belonging to the monoterpene hydrocarbons class (27.4%), followed by *γ*-terpinene (6.9%). The sample was practically devoid of oxygenated terpenes (0.3%) whereas moderate quantity of sesquiterpene hydrocarbons (5.4%) was observed, with *β*-caryophyllene (1.5%) as main representative.

Regarding to the composition of the EOs obtained from other accessions of *A. sicula* of different geographic origin, three articles have been published and concern plants collected in different part of Sicily [17,18] and in Algeria [16]. These results indicated the occurrence of three different chemotypes: myristicin-chemotype [17], apiole chemotype [18], and germacrene B-chemotype [16]. **As** clearly belongs to myrsticin chemotype.

As for the other *Athamanta* taxa myristicn has been identified as the main constituent of the aerial parts, and separate organs (flowers and fruits) of both *Athamanta turbith* ssp. *haynaldii* (Borbas & Uechtr.) Tutin [19,20,21,22,23], and *Athamanta turbith* ssp. *hungarica* (Borbas) Tutin [21], althoght the roots of both plants showed to be rich also of apiole [22]. A totally different profile has been observed in the sample obtained from the Greek aerial parts of *A. macedonica* (L.) Sprengel [15] that showed, as main metabolite, large extent of sabinene (50.5%), being totally devoid of phenylpropanoids.

In order to confirm the GC–MS identification of myristicin, and to test its biological properties, it was decided to isolate it by static chromatography and to confirm its structure by ^1^H- (Figure S1) and ^13^C-NMR (Figure S2) (Nuclear Magnetic Resonance).

The ^13^C-NMR spectrum showed 11 distinct carbon signals. The signal at 40.2 (CH_2_, C-1), the peak at 56.5 ppm, indicative of a methoxy group (-OCH_3_), and the one at 101.2 ppm, a methylenedioxy group (O-CH_2_-O), composed the aliphatic signal region. The signals at 115.8 (C-3) and 137.3 (C-2) were indicative of a double bond such as that present in the propylene chain linked at C-1. Finally, all the other carbon signals (102.7, 107.7, 133.5, 134.6, 143.5, and 148.8) referred to the carbons of the aromatic ring and confirmed, together with the analysis of proton signals reported in Section 4.4., the structure of myristicin (**1**).

### 2.2. Antimicrobial Properties

The antimicrobial activity of **As** was evaluated by determining the minimum inhibitory concentration (MIC) against all selected strains, including both Gram-positive and Gram-negative bacteria. In addition to the model strains *E. coli* and *S. aureus*, the panel included three other Gram-positive species (*B. subtilis*, *L. monocytogenes*, and *M. smegmatis*), two Gram-negative species (*P. aeruginosa* and *S. typhimurium*), and the yeast *C. albicans*, in order to obtain a comprehensive antimicrobial profile of **As** and its main constituents, myristicin and *β*-phellandrene, against microorganisms representative of intestinal and epithelial microbiota.

As shown in Table 2, **As** exhibited a broad-spectrum antimicrobial activity, with MIC values ranging from 24 to 30 mg/mL. The lowest MIC values were observed against Gram-negative strains (*S. typhimurium*, 24 mg/mL; *P. aeruginosa* and *E. coli*, 25 mg/mL), while slightly higher values were recorded for Gram-positive bacteria.

The individual constituents displayed distinct activity profiles. Myristicin showed markedly higher antimicrobial potency compared to *β*-phellandrene, with MIC values between 14 and 20 mg/mL, while *β*-phellandrene exhibited MICs of 29–30 mg/mL or higher. In particular, myristicin demonstrated strong inhibition against *E. coli* (MIC = 15 mg/mL) and *P. aeruginosa* (MIC = 14 mg/mL), confirming its major contribution to the overall activity of the essential oil.

These findings suggest that the antimicrobial potential of **As** is mainly attributable to the high content of myristicin, which likely acts synergistically with minor components to enhance bacterial growth inhibition. Nevertheless, definitive confirmation would require testing the oil depleted of myristicin.

### 2.3. Study on the Antimicrobial Mechanism of Action

To elucidate how **As** and its major constituents exert their antimicrobial activity, it was investigated their interaction with bacterial membranes and their potential to alter membrane integrity. Several studies have suggested that EO components rich in esters and monoterpenes can compromise the structural integrity of bacterial outer membranes (OM), increase permeability and ultimately leading to cell lysis [29]. To explore this mechanism, the hydrophobic fluorescent probe *N*-phenyl-1-naphthylamine (NPN) was employed. NPN exhibits minimal fluorescence in aqueous environments but shows a marked increase in fluorescence upon insertion into the hydrophobic regions of disrupted bacterial membranes. When the OM remains intact, NPN is excluded from the phospholipid bilayer; however, when the OM is damaged (of Gram-negative bacteria) it intercalates into lipid tails, resulting in a sharp rise in fluorescence intensity [30].

**Figure 4:**
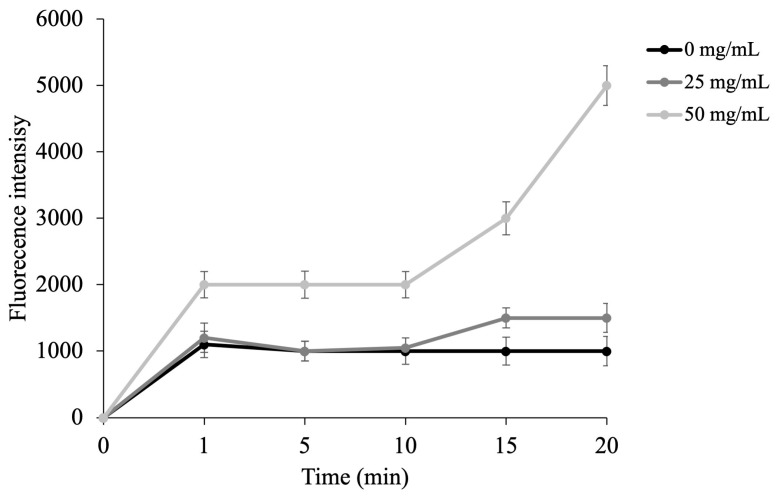
Outer Membrane Damage Assessed by NPN Assay. NPN fluorescence was used to evaluate outer membrane permeability in *E. coli* after treatment with MIC and 2× MICs of **As**. Data represent the mean of three independent experiments.

As shown in Figure 4, *E. coli* cells exposed to **As** at 25 mg/mL MIC displayed a moderate increase in fluorescence compared to the untreated control, indicating limited membrane perturbation. Conversely, treatment with **As** at twice the MIC (50 mg/mL) caused a pronounced and progressive enhancement of fluorescence intensity over time, reaching values more than double those of the control. This pattern suggests a dose-dependent destabilization of the outer membrane in *E. coli*, consistent with a membrane-targeting mode of action.

**Figure 5:**
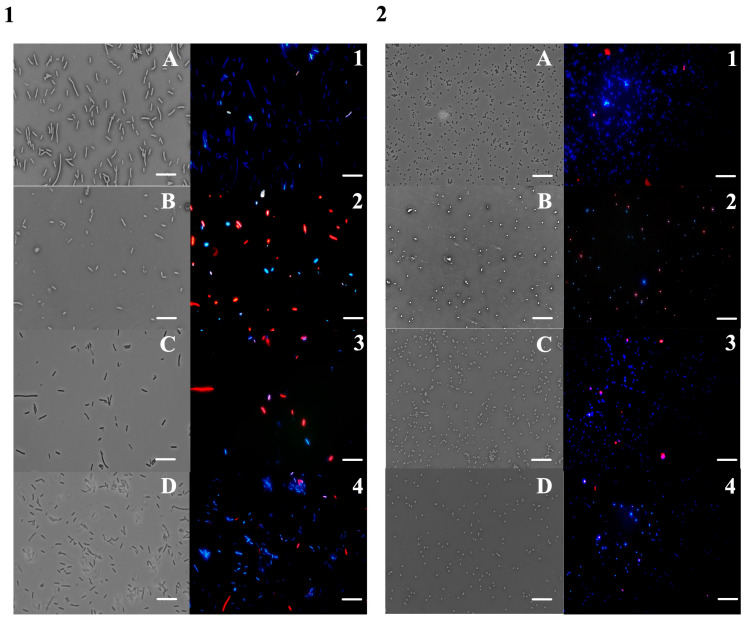
Fluorescence microscopy of *E. coli* (**panel 1**) and *S. aureus* (**panel 2**) cells after 30 min of treatment with **As** (**B**,**2**), myristicin (**C**,**3**) and *β*-phellandrene (**D**,**4**), stained with DAPI (blue) and propidium iodide (red). Untreated control cells are shown in (**A**,**1**). Scale bars: 5 µm.

These findings support the hypothesis that the antimicrobial effect of **As**, likely mediated by its major constituent myristicin, is associated with alterations in membrane integrity and increased permeability. To further confirm this observation and to assess potential damage to the inner membrane, additional fluorescence microscopy experiments using DAPI/PI double staining were performed. This approach allowed a more precise evaluation of membrane integrity in both Gram-negative and Gram-positive bacterial cells, providing a more comprehensive understanding of the antimicrobial mechanism. Specifically, the dual DAPI/propidium iodide (PI) method was performed against *E. coli* and *S. aureus*, representing Gram-negative and Gram-positive model strains, respectively, were incubated for 30 min at their corresponding MIC values of **As**, myristicin, and *β*-phellandrene. As shown in Figure 5, *E. coli* cells (**panel 1**) exhibited distinct fluorescence patterns depending on the treatment. Untreated cells (**A**,**1**) and those exposed to *β*-phellandrene (**D**,**4**) showed only blue fluorescence, indicating intact cell membranes and normal uptake of DAPI. In contrast, samples treated with **As** (**B**,**2**) and myristicin (**C**,**3**) displayed an intense red fluorescence due to PI uptake, consistent with significant membrane damage and loss of membrane integrity. This result confirms that both **As** and myristicin induce disruption of the bacterial envelope, leading to increased permeability and cell death. The fluorescence behaviour of *S. aureus* cells (**panel 2**) differed from that observed for *E. coli*. All treatments primarily exhibited blue fluorescence, suggesting that the Gram-positive cell wall provided higher resistance to membrane disruption. However, samples treated with **As** displayed faint red fluorescence, indicating partial membrane compromise at the MIC.

These observations align well with the MIC and NPN uptake results. The greater susceptibility of *E. coli* compared to *S. aureus* supports the hypothesis that **As** primarily targets the bacterial membrane, particularly in Gram-negative strains where the outer membrane plays a crucial role in permeability. The strong activity of myristicin further confirms its central role in the overall antimicrobial potential of the EO, likely due to its ability to perturb lipid bilayers and destabilize the cell envelope structure.

### 2.4. Antibiofilm Activity of As

After identifying the bacterial target of **As**, it was investigated its antibiofilm properties. The assay was performed on *Mycobacterium smegmatis*, a model organism widely used to study biofilm formation and maturation, to assess the ability of **As** to interfere with organized microbial growth and persistence, processes closely related to the antimicrobial activity previously observed against skin and gut-associated bacterial strains [31].

Given that the minimum inhibitory concentration (MIC) of **As** against *M. smegmatis* was previously determined to be 30 mg/mL, sub-MICs (0–1 mg/mL) were tested to evaluate the ability of the EO to inhibit biofilm formation.

**Figure 6:**
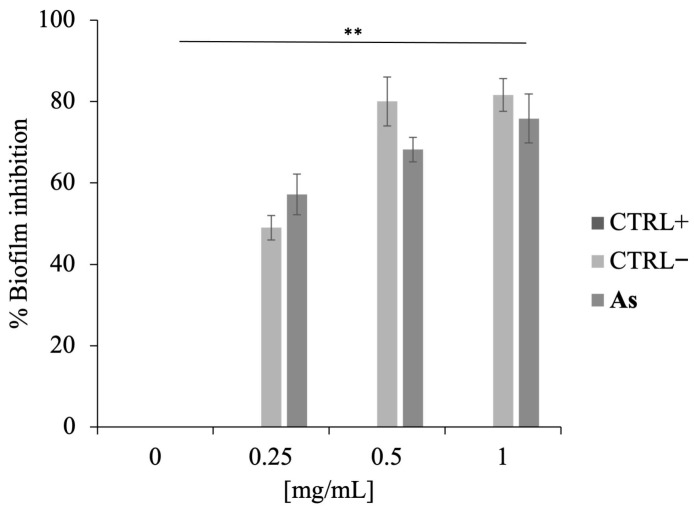
Inhibition of biofilm formation by **As** on *M. smegmatis*. Biofilm biomass was quantified after treatment with sub-MICs (0–1 mg/mL) of **As**. CTRL+ cells were untreated, CTRL− cells were treated with kanamycin. Data represent the average of three independent experiments. Statistical analysis was performed relative to the control by two-tailed paired *t*-test. *p*-value (** *p* < 0.01).

As shown in Figure 6, **As** exhibited a dose-dependent inhibition of biofilm development. At the concentration of 1 mg/mL, biofilm formation was reduced by approximately 80%, indicating a strong inhibitory effect even at subinhibitory levels.

This finding is particularly relevant, as it suggests that **As** can interfere with the early stages of biofilm establishment, thereby potentially preventing bacterial colonization and persistence, key factors in chronic infections.

### 2.5. Antioxidant Activity of As and Single Components

It is well established in the literature that EOs, in addition to exhibiting remarkable antimicrobial properties, can also display significant antioxidant potential. In light of this evidence, the antioxidant activity of **As** and its main constituents, myristicin and *β*-phellandrene, was evaluated.

**Figure 7:**
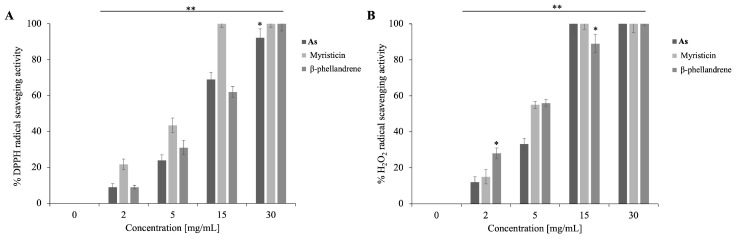
Antioxidant activity of **As**, Myristicin and *β*-phellandrene. (**A**): DPPH assay; (**B**): H_2_O_2_ scavenging assay. **As**, Myristicin and *β*-phellandrene were tested at concentrations ranging from 0 to 30 mg/mL. Data represent the average of three independent experiments. Statistical analysis was performed relative to the standard by two-tailed paired *t*-test. *p*-value (* *p* ≤ 0.05; ** *p* < 0.01).

As shown in Figure 7, the free radical scavenging activity was determined using two complementary assays: the DPPH test, which assesses the electron-donating ability of antioxidant compounds, and the hydrogen peroxide (H_2_O_2_) scavenging assay, which measures the capacity to neutralize reactive oxygen species (ROS) such as H_2_O_2_.

In Figure 7A (DPPH assay), all samples exhibited a marked, dose-dependent increase in antioxidant activity. Myristicin displayed the highest efficacy, reaching almost 100% inhibition between 10 and 15 mg/mL, while *β*-phellandrene was considerably less active, achieving similar values only at higher concentrations (30 mg/mL). **As** showed an intermediate trend, suggesting possible interactions among the various components that could modulate the overall antioxidant potential compared to the single compound. Figure 7B (H_2_O_2_ scavenging assay) revealed a similar but more pronounced effect. Both **As** and myristicin achieved complete (100%) scavenging of hydrogen peroxide at 15 mg/mL, indicating a stronger dose-dependent response compared to the DPPH assay. *β*-Phellandrene again exhibited lower activity, reflecting its limited ability to interact with ROS.

To quantify the antioxidant activity of the samples comparatively, the IC_50_ values (concentration required to achieve 50% inhibition of radical activity) were calculated, as shown in Table 3. The results confirm that myristicin has the highest antioxidant potency, followed by As, while *β*-phellandrene is significantly less active in both tests.

Overall, these findings indicate that the antioxidant activity of **As** is mainly attributable to myristicin, with methoxy and methylenedioxy groups enable stabilization of free radicals through electron donation and charge delocalization mechanisms. The EO therefore demonstrates a strong dose-dependent antioxidant effect in both assays, supporting its biological relevance as a source of bioactive compounds with antimicrobial, antibiofilm, and antioxidant properties in vitro.

### 2.6. Cytotoxic Activity of As and of Its Individual Compounds

**Figure 8:**
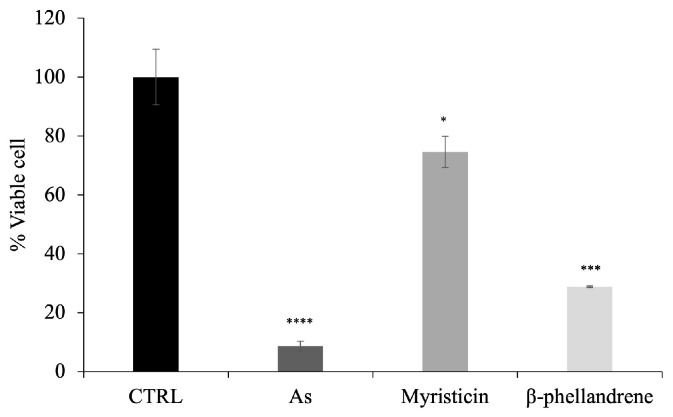
Cytotoxicity of **As**, Myristicin and *β*-phellandrene on HaCaT cells assessed by MTT assay. Cell viability was evaluated after 24 h of treatment. Cells were treated with the highest concentrations corresponding to the MIC values for each compound (30 mg/mL for **As** and *β*-phellandrene, and 15 mg/mL for Myristicin). Data are expressed as percentage of viable cells relative to untreated control (CTRL) and are presented as mean of three independent experiments. Statistical analysis was performed using a two-tailed paired *t*-test relative to untreated cells: * *p* < 0.05*,* *** *p* < 0.001, **** *p* < 0.0001.

To evaluate the potential cytotoxicity of **As** on eukaryotic cells, human HaCaT epithelial cells were used in an MTT assay. The assay was performed by testing the essential oil and the individual components at the highest concentrations corresponding to their respective MIC values. The results showed a reduction in cell viability after 24 h of incubation with ***As***, myristicin, and *β*-phellandrene, as shown in Figure 8.

In particular, treatment with **As** (30 mg/mL) resulted in a marked reduction in cell viability, with values less than 20% compared to the untreated control, indicating a cytotoxic effect. When cells were treated with individual components of the oil, specifically compounds **1** and **2**, differential effects were observed. A significant cytotoxic effect was also observed following treatment with *β*-phellandrene (30 mg/mL), which reduced cell survival to approximately 25–30%.

In contrast, myristicin, tested at a concentration of 15 mg/mL, showed a more moderate cytotoxic effect, maintaining cell viability approximately 80% compared to the control. These results indicate that, at the concentrations tested, myristicin is the least toxic compound against HaCaT cells. Overall, the data demonstrate differential cytotoxicity of the tested compounds after 24 h of exposure, with a toxicity gradient following the order **As** > *β*-phellandrene > myristicin, suggesting a more favorable cellular safety profile for the latter at biologically active concentrations. Notably, the whole EO (**As**) exerted a substantially greater cytotoxic effect than either myristicin or *β*-phellandrene alone, suggesting a possible synergistic or additive interaction among its constituents contributing to the overall reduction in cell viability.

Indeed, it is well known in the literature that myristicin, when evaluated as a single compound, exhibits a generally moderate cytotoxicity profile that is highly dependent on the dose, exposure time, and cellular model used. As reported in the review by Seneme et al. [28], the cytotoxic effects of myristicin are more evident in tumor cell lines, where it can induce a reduction in cell viability and activation of apoptotic pathways, while in non-tumor cells the molecule often shows better tolerability, with reductions in viability observed mainly at high concentrations or under specific experimental conditions. Furthermore, the same review highlights that a significant portion of the toxicity attributed to myristicin is associated with its bioactive metabolites, rather than the parent molecule, suggesting that pure myristicin may exert a more limited cytotoxic impact. In this context, our results obtained on HaCaT cells after 24 h of treatment, showing a limited reduction in cell viability in the presence of myristicin compared to **As** and *β*-phellandrene, are fully consistent with what reported in the literature and support the hypothesis of a more favorable cellular safety profile for myristicin at the biologically active concentrations tested.

### 2.7. Antioxidant Activity of As on Eukaryotic Cells

**Figure 9:**
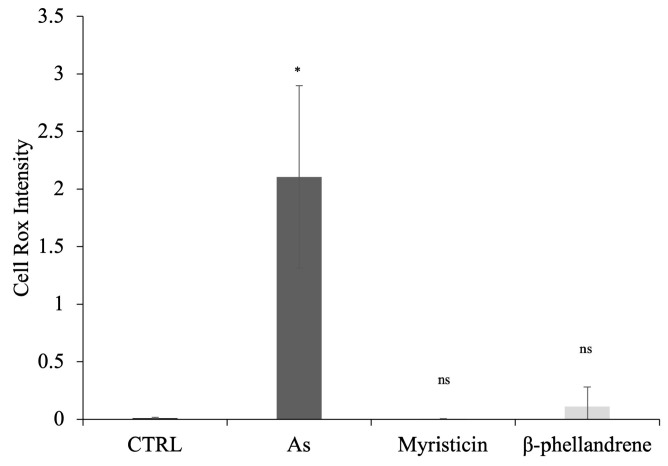
Effect of **As**, myristicin and *β*-phellandrene on intracellular ROS generation in HaCaT cells. Oxidative stress was evaluated using the CellROX™ fluorescent probe. HaCaT cells were treated with **As** (30 mg/mL), myristicin (15 mg/mL) and *β*-phellandrene (30 mg/mL) for 24 h before ROS detection. Data are expressed as relative fluorescence intensity and are presented as mean of three independent experiments. Statistical analysis was performed using a two-tailed paired *t*-test relative to the untreated control (CTRL); ns (not significant); * *p* < 0.05.

A fluorescent dye (CellRox) sensitive to Intracellular reactive oxygen species (ROS) was used to assess the production of reactive oxygen species in human HaCaT epithelial cells after 24 h of treatment with **As**, myristicin, and *β*-phellandrene at concentrations corresponding to their higher respective MIC values. As shown in Figure 9, treatment with **As** (30 mg/mL) resulted in a significant increase in intracellular ROS levels compared to the untreated control, indicating significant increase in ROS production, and pronounced oxidative stress.

In contrast, cells treated with myristicin (15 mg/mL) did not show a significant increase in ROS production compared to the control, suggesting that this compound does not induce oxidative stress at the biologically active concentrations tested. A slight increase in ROS levels was observed following treatment with *β*-phellandrene (30 mg/mL); however, this increase was significantly lower than that induced by **As**.

These results indicate that the cytotoxicity observed for **As** is associated with a ROS-dependent mechanism, while myristicin exerts its effects without promoting the generation of reactive oxygen species, further supporting its cellular safety profile in HaCaT cells. Consistent with the literature, myristicin has been described as a compound capable of exerting moderate cytotoxic effects independent of oxidative stress, particularly in non-tumor cell models [32].

## 3. Discussion

The present study provides a comprehensive overview of the phytochemical composition and biological activities of **As**, highlighting its antimicrobial, antibiofilm, and antioxidant potential. The antimicrobial profile obtained through MIC determination revealed that **As** exhibited broad-spectrum inhibitory activity, with a higher efficacy against Gram-negative bacteria. Among the individual components, myristicin demonstrated the most potent effect, followed by *β*-phellandrene, whose activity was markedly lower.

These findings align with previous studies on *Apiaceae* EOs, which frequently report that phenylpropanoid- and monoterpene-rich oils display antimicrobial activity through mechanisms involving membrane perturbation and leakage of intracellular contents [33,34]. Comparable results have been described for *Pimpinella anisum* and *Pastinaca sativa* EOs, where lipophilic constituents were shown to intercalate into bacterial membranes, increasing permeability and leading to cell death [35,36].

The NPN uptake assay performed in this study supports this mechanism, revealing enhanced fluorescence intensity upon exposure to **As**, particularly at concentrations above the MIC, indicative of outer membrane destabilization. The effect was concentration-dependent, suggesting that the EO compromises the permeability barrier of Gram-negative bacteria such as *E. coli*. These results are consistent with literature reports showing that EOs rich in monoterpenes and phenylpropanoids induce similar permeabilization effects by interacting with membrane lipids [31,37]. Fluorescence microscopy using DAPI/PI double staining further confirmed the membrane-disrupting potential of **As** and myristicin. The intense red fluorescence observed in treated *E. coli* cells demonstrated clear inner membrane damage and loss of viability, whereas *S. aureus* cells displayed mainly blue fluorescence, indicating higher structural resistance. These findings, in agreement with MIC and NPN results, suggest that **As** acts primarily by targeting bacterial membranes, a mechanism more effective against Gram-negative strains due to the easier penetration of lipophilic compounds into the outer membrane.

The differential antimicrobial activity observed between Gram-negative and Gram-positive bacteria can be explained by the chemical nature and membrane interactions of myristicin, the main phenylpropanoid identified in **As** (62.2%).

Myristicin is a neutral and moderate lipophilic molecule that can readily partition into lipid bilayers. Its aromatic ring, substituted with methoxy and methylenedioxy groups, confers low polarity and high membrane affinity, enabling efficient penetration into hydrophobic environments. Recent studies demonstrated that myristicin and related phenylpropanoids alter bacterial membrane organization and increase permeability, leading to leakage of intracellular components and loss of cell integrity [38].

Because it is uncharged, myristicin is not repelled by the negatively charged surface components typical of Gram-negative bacteria, such as lipopolysaccharides (LPS). This facilitates its access to the outer membrane, where it can intercalate among phospholipid tails. Once inserted, its hydrophobic moiety perturbs the bilayer structure, promoting membrane destabilization and enhanced permeability, as confirmed in our study by NPN uptake and DAPI/PI fluorescence assays. These effects are consistent with molecular observations reported for other phenylpropanoids, which can disrupt lipid–lipid packing via π–π and van der Waals interactions within the hydrophobic core [32].

In contrast, the thick peptidoglycan layer of Gram-positive bacteria acts as a rigid structural barrier that limits the diffusion of nonpolar compounds such as myristicin [39]. Moreover, Gram-positive species lack an outer lipid membrane, reducing the number of hydrophobic sites where such lipophilic molecules can exert their destabilizing effect [40]. This structural difference explains the lower susceptibility observed for Gram-positive strains.

The neutral charge and balanced lipophilicity of myristicin therefore provide an optimal profile for selective interaction with the lipid-rich outer membrane of Gram-negative bacteria, supporting its higher antimicrobial efficacy.

Conversely, *β*-phellandrene, a highly hydrophobic monoterpene hydrocarbon devoid of polar functional groups, shows limited insertion into lipid bilayers and weak interactions with membrane phospholipids or proteins. As a result, it exerts minimal membrane-disrupting activity, consistent with its low antimicrobial potency and the negligible fluorescence signal observed in DAPI/PI assays.

Overall, these findings indicate that the antimicrobial efficacy of **As** is primarily driven by the amphiphilic and uncharged nature of myristicin, which allows membrane interaction and perturbation, particularly in Gram-negative species. The combined presence of myristicin and minor volatile constituents likely enhances this effect through additive or synergistic interactions, a well-known feature of complex EO systems.

Following the investigation of the antimicrobial mechanism, the antibiofilm activity of **As** was evaluated using *Mycobacterium smegmatis*, a well-established model organism for studying biofilm formation and maturation [31]. The oil displayed a strong inhibitory effect, reducing biofilm biomass by approximately 80% at 1 mg/mL, a concentration well below its MIC. This result suggests that **As** interferes with the initial stages of biofilm development, possibly by altering cell adhesion and matrix formation. Given the structural similarities between *M. smegmatis* and clinically relevant pathogens capable of forming persistent biofilms (e.g., *S. aureus*, *P. aeruginosa*), this finding supports the potential of **As** as antibiofilm agent.

In addition to its antimicrobial and antibiofilm effects, **As** demonstrated a remarkable antioxidant potential. Both DPPH and H_2_O_2_ assays revealed dose-dependent scavenging activity, with myristicin showing the highest efficacy and *β*-phellandrene the lowest. Notably, **As** and myristicin reached nearly complete radical scavenging at 15 mg/mL in both assays. The calculated IC_50_ values confirmed the superior activity of myristicin (6.2 and 7.1 mg/mL for DPPH and H_2_O_2_, respectively) compared to **As** (8.5 and 7.8 mg/mL) and *β*-phellandrene (≈15 mg/mL). The antioxidant potential of **As** can thus be primarily attributed to the electron-donating and resonance-stabilizing abilities of myristicin, whose oxygenated substituents allow delocalization of the unpaired electron.

The strong radical-scavenging capacity of **As**, together with its antimicrobial and antibiofilm properties, highlights its multifunctional biological potential.

However, antioxidant activity measured in cell-free assays, such as DPPH and H_2_O_2_ scavenging, does not necessarily translate into intracellular redox modulation. While these assays reflect the intrinsic radical-scavenging capacity of the compounds, cellular systems involve complex metabolic, membrane-related, and mitochondrial processes. Accordingly, at MIC-associated concentrations, **As** induced increased intracellular ROS levels and a marked reduction in HaCaT cell viability, indicating that strong chemical antioxidant activity may coexist with dose-dependent cytotoxic responses at the cellular level. In contrast, myristicin displayed a more favorable safety profile, causing only a moderate reduction in cell viability without triggering oxidative stress, thus suggesting a ROS-independent mechanism of action. These combined effects could be particularly relevant for skin- and intestinal applications, where oxidative stress and microbial imbalance frequently coexist. Similar observations have been reported for Apiaceae derived EOs employed in natural antioxidant and antimicrobial formulations [34,41].

Taken together, these findings suggest that the biological activity of **As** is largely driven by its high myristicin content, which emerges as the principal contributor to the observed antimicrobial, antibiofilm, and antioxidant effects. While the whole EO exhibits strong antimicrobial and radical-scavenging properties, its cytotoxic and pro-oxidant effects at MIC-associated concentrations highlight the importance of component-specific evaluation. In this context, myristicin stands out as a key bioactive molecule, combining potent antimicrobial efficacy with a more favorable safety profile in HaCaT cells and a ROS-independent mechanism of action.

These features support the potential of myristicin as a promising natural antimicrobial agent, particularly for skin and intestinal applications, where microbial control must be balanced with cellular compatibility. Therefore, myristicin from *A. sicula* L. represents a valuable candidate for the development of targeted cosmetic, nutraceutical, and pharmacological formulations, either as a purified compound or as a rationally enriched component of EO-based systems.

## 4. Materials and Methods

### 4.1. Plant Material

Aerial parts (flowers, leaves, and stems) of *Athamanta sicula* L. were collected in the area near Contrada Pioppo, Caltabellotta (AG), Sicily, Italy, 550 m a.s.l. (13.168519 E, 37.577449 N); habitat: cracks on a limestone rock. One of the samples, identified by Prof. Giuseppe Bazan, has been stored in the Herbarium Mediterraneum Panormitanum (PAL), (Voucher N.109808), of the Botanical Garden of the University of Palermo, Italy.

### 4.2. Extraction of EO

Fresh aerial parts (flowers, leaves, and stems, total 5 kg) were subjected to hydrodistillation for 3 h, according to the standard procedure described in European Pharmacopoeia [42]. Samples yielded 0.15% of EO.

### 4.3. GC-MS Analysis

Analysis of EO was carried out according to the procedure reported by Lauricella et al. [43]. Linear retention indices (LRIs) were calculated using a mixture of pure *n*-alkanes (C_8_–C_40_), and all the peaks’ compounds were identified by comparison with MS and by comparison of their relative retention indices with WILEY275, NIST 17, ADAMS, and FFNSC2 libraries.

### 4.4. Isolation of Myristicin, Spectroscopical Analysis (NMR)s and Chemicals

The EO (500 mg) was fractionated, using a flash silica gel column (Biotage^®^ Sfär Silica HC D, High-Capacity Duo 20 μm, 25 g, Biotage AB, Uppsala, Sweden). Columns were typically run using a gradient method, beginning with 100% petroleum ether and gradually increasing the polarity using ethyl acetate. Fractions of 8 mL were collected and analyzed by TLC and GC-MS analysis. The fractions which contained desired terpenoid were combined. A 99:1 (*v*/*v*) petroleum ether-ethyl acetate mixture was used for isolation of myristicin (223 mg). The purity of the final products was determined by GC/MS analysis, and the identity of the compound was confirmed by ^1^H and ^13^C-NMR. The NMR spectra were recorded on a Bruker Avance II instrument (400 MHz for ^1^H-NMR and 100 MHz for ^13^C-NMR, Bruker, Billerica, MA, USA). Deuterated chloroform (CDCl_3_) was used as solvent with the peaks at 7.27 and 77.0 ppm as references for ^1^H- and ^13^C-NMR spectra, respectively. *β*-Phellandrene (CAS No.: 555-10-2) was purchased from Sigma-Aldrich (Merk Life Science S.r.l., Via Monte Rosa, 93, 20149 Milan, Italy).

#### Myristicin (**1**)

Myristicin (**1**, Figure 3), yellow oil, C_11_H_12_O_3_: MS: 192 (M+, 100%), 165 (25%), 119 (34%), 91 (48%), 77 (27%), 65 (34%) ^1^H-MNR (500 MHz, CDCl_3_) (Figure S1): 3.30 (2H, d, *J* = 6.5 Hz, 1-C*H_2_*), 3.90 (3H, s, 3′-OC*H_3_*), 5.09 (2H, m, 3-C*H_2_*), 5.92 (1H, m, 2-H), 5.94 (2H, s, O-C*H_2_*-O), 6.36 (1H, s, 6′-H), 6.39 (1H, s, 2′-H); ^13^C-NMR (100 MHz, CDCl_3_) (Figure S2): 40.2 (C-1), 56.5 (C-O*C*H_3_), 101.2 (O-*C*H_2_-O), 102.7 (C-6′), 107.7 (C-2′), 115.8 (C-3), 133.5 (C-1′), 134.6 (C-4′) 137.3 (C-2), 143.5 (C-3′), 148.8 (C-5′). ^1^H- and ^13^C-NMR spectra are presented in Figures S1 and S2. Its NMR data were in accord with the reported data of You et al. [44].

### 4.5. Bacterial Strains

The antimicrobial activity of **As**, myristicin and *β*-phellandrene was evaluated against different strains Gram-negative such as: *Escherichia coli* DH5*α*, *Pseudomonas aeruginosa* PAO1 ATCC15692 and *Salmonella typhimurium* ATCC14028; Gram-positive such as: *Staphylococcus aureus* ATCC6538P, *Bacillus subtilis* AZ54, *Listeria monocytogenes* ATCC 19115 and *Mycobacterium smegmatis* MC^2^155 and the yeast *Candida albicans* ATCC14053. *E. coli* and *S. aureus* were used as model strains.

### 4.6. Determination of Minimum Inhibitory Concentrations (MIC)

The antimicrobial activity of **As**, myristicin, and *β*-phellandrene was evaluated by determining the minimum inhibitory concentrations (MICs) against selected bacterial strains using the broth microdilution method, according to CLSI guidelines [45].

Bacterial cultures (5 × 10^5^ CFU/mL), grown overnight, were added to 96-well microplates containing 195 μL of Mueller–Hinton broth (CAM-HB; Difco Becton Dickinson, Franklin Lakes, NJ, USA). Samples diluted in 50% DMSO were tested at concentrations ranging from 1 to 30 mg/mL, based on the relative proportion of each component in the EO. Plates were incubated at 37 °C for 24 h.

The MIC was assessed by measuring the optical density at 600 nm using a microplate reader, defined as the lowest sample concentration that completely inhibited visible growth at OD 600 nm. Untreated cells served as negative controls, whereas ampicillin and 50% DMSO were used as positive controls.

To confirm MIC results, 15 μL of resazurin solution (0.01%) was added to each well, followed by incubation at 37 °C for 30 min. The redox-dependent color change was recorded by measuring absorbance at 600 nm.

### 4.7. N-Phenyl Naphthylamine (NPN) Assay

The ability of **As** to permeabilize the bacterial outer membrane (OM) was assessed by means of the 1-N-phenylnaphthylamine (NPN) uptake assay, adapted from the method described by Jia et al. [46] with minor modifications.

*E. coli* cultures were incubated overnight under standard growth conditions, then collected by centrifugation, rinsed with PBS, and resuspended in 5 mM HEPES buffer (pH 7.2) until reaching an optical density of 0.5 ± 0.02 at 600 nm. For each experimental condition, 50 µL of the test sample (used at concentrations corresponding to the MIC and twice the MIC) was mixed with 100 µL of the bacterial suspension in black 96-well fluorescence microplates. Subsequently, 50 µL of a 40 µM NPN solution was added. Control wells consisted of bacterial cells and NPN in HEPES buffer without *As*. Fluorescence was measured at excitation and emission wavelengths of 350 nm and 420 nm, respectively, using a microplate fluorimeter. The increase in fluorescence intensity was used as an indicator of OM permeabilization.

### 4.8. Fluorescence-Based Viability Assay Using DAPI and PI

The integrity of the bacterial inner membrane (IM) was evaluated by fluorescence microscopy using DAPI (4′,6-diamidino-2-phenylindole dihydrochloride; Sigma-Aldrich, Milan, Italy) and propidium iodide (PI; Sigma-Aldrich, Milan, Italy), following the method described by Di Girolamo et al. [47].

Briefly, 100 μL of *E. coli* and *S. aureus* suspensions were incubated at 37 °C for 4 h under shaking, in the presence or absence of **As**, myristicin, or *β*-phellandrene, at their respective MIC values (for *E. coli*: 25, 16, and 30 mg/mL; for *S. aureus*: 30, 15, and 30 mg/mL). Following incubation, 10 μL of each sample was combined with a staining solution containing DAPI (1 μg/mL) and PI (20 μg/mL) and incubated in the dark. Fluorescence images were obtained using an Olympus BX51 microscope (Olympus, Tokyo, Japan) equipped with a filter set specific for DAPI detection (excitation/emission: 358/461 nm). The exposure time for DAPI/PI double staining was fixed at 1000 ms. Image acquisition was performed using an Olympus DP70 digital camera (Tokyo, Japan).

### 4.9. Inhibition of Biofilm Development Assays

The antibiofilm activity of **As** was evaluated against *Mycobacterium smegmatis* biofilms using the crystal violet (CV) colorimetric assay, as described by Di Napoli et al. [48].

Biofilms were grown in 24-well plates and incubated at 37 °C for 3 days with or without **As** at concentrations ranging from 0 to 1 mg/mL. Untreated cells were used as positive controls, while kanamycin-treated cultures served as negative controls.

Following incubation, the biofilm biomass was stained with crystal violet, and absorbance was measured at 570 nm using a Multiskan microplate reader (Thermo Electron Corporation, Waltham, MA, USA). To correct for differences in planktonic growth, results were normalized by calculating the ratio between OD_570_ (biofilm biomass) and OD_600_ (planktonic cell density). The percentage of biofilm inhibition was then determined by comparing the normalized OD values of treated samples to those of untreated controls according to the following formula:

Biofilm formation (%) = [(OD_570_/OD_600_)treated/(OD_570_/OD_600_)control] × 100

where (OD_570_/OD_600_)treated represents the normalized biofilm value in the presence of **As** or antibiotics; (OD_570_/OD_600_)control represents the normalized biofilm value of the untreated control; and

Biofilm inhibition (%) = (100 − % Biofilm formation).

### 4.10. DPPH and H_2_O_2_ Scavenging Capacity Assay

The antioxidant capacity of **As** and its individual components, was evaluated through two complementary assays: DPPH radical scavenging and hydrogen peroxide scavenging, following the methodology described by Napolitano et al. For both assays, a range of concentrations (0–30 mg/mL) was prepared in a final reaction volume of 1 mL [49].

In the DPPH assay, a fresh prepared 0.1 mM DPPH solution in 100% methanol was adjusted to an initial absorbance of ≤1.0. Samples were incubated with the DPPH solution at room temperature for 30 min, after which the decrease in absorbance at 517 nm was measured

For the hydrogen peroxide scavenging assay, fresh hydrogen peroxide solution (50 mM potassium phosphate buffer, pH 7.0; 0.036% *w*/*w* H_2_O_2_) was used. Samples at varying concentrations were incubated with 1 mL of this solution at room temperature for 30 min. After centrifugation to remove any particulate matter, the absorbance of the supernatant was measured at 240 nm to assess the residual hydrogen peroxide. The scavenging percentage was determined by

H_2_O_2_ and DPPH scavenged (%) = (1 − Abs/AC) × 100

where AC represents the absorbance of the solution without the sample, and Abs is the absorbance following incubation with the sample. To account for turbidity and potential physical quenching due to the immiscibility of the EO in methanol, each sample was first read before DPPH addition (T_0_), and again after 30 min of incubation (T_30_). The corrected absorbance value (AS) was calculated as T_30_–T_0_, ensuring the removal of background absorbance caused by the oil phase.

As positive control ascorbic acid was included treated under the same experimental conditions.

Together, these assays provided a comprehensive evaluation of the antioxidant potential against both stable free radicals and reactive oxygen species.

### 4.11. Eukaryotic Cell Culture

HaCaT (human epidermal cells), purchased from CLS Cell Lines Service, Eppelheim, Germany, were maintained in Dulbecco’s Modified Eagle Medium (DMEM) (61965-026, GIBCO, Grand Island, NY, USA) supplemented with 10% FBS (Euroclone, ECS500L, Pero, Italy) 1%, Penicillin-Streptomycin (ECB3001D, Euroclone, Pero, Italy), 4mM L-Glutamine (ECB3000D, Euroclone, Pero, Italy). Cells grew at 37 °C in a humid atmosphere of 5% (*v*/*v*) CO_2_.

### 4.12. MTT

28,000 HaCaT cells/well were seeded into 96-well plates. 24 h after seeding, cells were treated with *Athamanta sicula*, Myristicin or *β*-phellandrene for 24 h, at the indicated concentrations. All compounds were dissolved in DMSO, and control cells were treated with the same final concentration of DMSO used in the compound-treated samples. Cell viability was then assessed by a standard MTT assay (CT01, Sigma-Aldrich, St. Louis, MO, USA). Briefly, cells were incubated for 3 h at 37 °C in the dark with media containing 0.5 mg/mL of MTT solution. Afterward, medium was replaced with isopropanol and incubated for another 30 min at 37 °C. Then absorbance at 570 and 620 nm was determined using EnVision plate reader (PerkinElmer, Waltham, MA, USA). Cell viability was calculated using the following formula:

Viability = (Normalized Mean Absorbance of Treated Cells/Normalized Mean Absorbance of Control Cells) × 100%

### 4.13. CellROX

HaCaT cells were seeded into 96-well plates. 24 h after seeding, cells were treated with **As**, myristicic, and *β*-phellandrene for 24 h, at the indicated concentrations, followed by CellROX Green Reagent fluorogenic probe detection for measuring oxidative stress in live cells (C10444, Invitrogen, Waltham, MA, USA). Cells were incubated for 30 min at 37 °C in the dark with media containing 5 mM of CellROX solution. Afterward, medium was replaced with fresh medium. Then absorbance at 488 nm was determined using CellDiscoverer CD7 (ZEISS, Oberkochen, Germany).

### 4.14. Statistical Analysis

Statistical analysis was performed using a two-tailed paired Student’s *t*-test, suitable for comparing related sample groups. The analysis was conducted in Microsoft Excel (Microsoft Office 365), and results were considered statistically significant when *p*-values were less or same than 0.05. Data are expressed as mean ± standard deviation (SD) from at least three independent experiments.

## 5. Conclusions

This study describes the chemical composition and biological activities of the EO obtained from the aerial parts of *Athamanta sicula* L., the only species of the genus *Athamanta* growing wild in Sicily. The EO was characterized by a high content of the phenylpropanoid myristicin (62.2%), together with lower amounts of *β*-phellandrene and *γ*-terpinene, defining a myristicin-rich chemotype distinct from other *A. sicula* accessions previously reported.

The EO and its main constituents were evaluated for antimicrobial, antibiofilm, and antioxidant activities, as well as for their mechanisms of action on bacterial membranes. The results demonstrated that myristicin is the main contributor to the antibacterial efficacy of the oil, particularly against Gram-negative bacteria, and to the strong inhibition of biofilm formation, likely through membrane-targeted mechanisms.

Cytotoxicity and oxidative stress assays performed on human keratinocyte HaCaT cells further highlighted differences between the whole EO and its individual components. While the EO showed strong antimicrobial and radical-scavenging properties, it also induced a marked reduction in cell viability and increased intracellular ROS levels at MIC-associated concentrations. In contrast, myristicin displayed a more favorable safety profile, causing only a moderate decrease in cell viability without inducing oxidative stress, suggesting a ROS-independent mechanism of action.

Overall, these findings identify myristicin as a key bioactive compound of *A. sicula*, combining potent antimicrobial and antibiofilm activity with improved cellular compatibility. This balance supports its potential use as an important natural antimicrobial agent, particularly for applications where efficacy must be coupled with host cell safety, such as skin- and intestinal-related contexts.

## Figures and Tables

**Table 1:** Chemical composition (%) of **As** collected at Caltabellotta (Sicily).

No.	Compounds ^a^	LRI ^b^	LRI ^c^	Area (%)
**1**	*β*-Thujene	922	920	0.1
**2**	*α*-Pinene	927	931	0.1
**3**	Sabinene	968	969	0.1
**4**	*β*-Pinene	989	982	1.1
**5**	*α*-Phellandrene	1003	998	1.0
**6**	*p*-Cymene	1023	1019	0.8
**7**	*β*-Phellandrene	1031	1032	12.2
**8**	*cis*-*β*-Ocimene	1038	1034	1.4
**9**	*trans*-*β*-Ocimene	1047	1044	1.0
**10**	*γ*-Terpinene	1060	1060	6.9
**11**	*α*-Terpinolene	1083	1083	1.8
**12**	Nonanal	1105	1102	0.1
**13**	*neo*-*allo*-ocimene	1128	1128	1.0
**14**	*trans*-2-Nonenal	1160	1163	0.1
**15**	4-Terpineol	1178	1173	0.1
**16**	*δ*-Elemene	1328	1331	0.4
**17**	*α*-Copaene	1370	1361	0.7
**18**	*β*-Caryophyllene	1413	1413	1.5
**19**	*α*-Bergamotene	1425	1431	1.0
**20**	*α*-Caryophyllene	1448	1445	0.3
**21**	Germacrene D	1475	1477	0.6
**22**	*γ*-Elemene	1489	1480	0.1
**23**	Zingiberene	1496	1487	1.0
**24**	Myristicin	1529	1530	62.2
**25**	Spathulenol	1583	1584	0.2
**26**	Caryophyllene oxide	1587	1581	0.1
**27**	*α*-Cadinol	1653	1634	0.2
**28**	Apiole	1667	1669	2.2
**29**	*α*-Bisabolol	1685	1682	0.1
	**Monoterpene Hydrocarbons**			**27.4**
	**Oxygenated Monoterpenes**			**0.1**
	**Sesquiterpene Hydrocarbos**			**5.4**
	**Oxygenated Sesquiterpenes**			**0.6**
	**Phenylpropanoids**			**64.4**
	**Aldehydes**			**0.2**
	**Total**			**98.1**

^a^ Components listed in order of elution on a DB-5 MS non-polar column. ^b^ Experimental LRIs on a DB-5 MS non-polar column. ^c^ LRIs based on the literature (https://webbook.nist.gov/, accessed on 22 December 2025).

**Table 2:** Minimum Inhibitory Concentrations (MICs) were determined by the broth microdilution method against Gram-positive and Gram-negative strains. Values represent the mean of three independent experiments and are expressed in mg/mL and ± standard deviation SD.

Strains	MIC [mg/mL] ± SD		
	As	Myristicin	*β*-Phellandrene
*E. coli*	25 ± 0.7	15 ± 0.9	30 ± 0.4
*P. aeruginosa*	25 ± 0.9	14 ± 0.7	30 ± 1.7
*S. typhimurium*	24 ± 1.1	15 ± 1.3	29 ± 1.1
*S. aureus*	30 ± 0.5	16 ± 0.9	30 ± 0.4
*B. subtilis*	26 ± 0.7	16 ± 0.5	30 ± 0.3
*L. monocytogenes*	29 ± 1.2	17 ± 0.3	>30
*M. smegmatis*	30 ± 0.3	20 ± 1	>30
*C. albicans*	25 ± 1	15 ± 0.5	30 ± 0.7

**Table 3:** IC_50_ values for the antioxidant activity of **As**, myristicin, *β*-phellandrene, and ascorbic acid (control) in the DPPH and H_2_O_2_ assays. Values represent the mean of three independent experiments and are expressed in mg/mL and ± standard deviation SD.

	IC_50_ [mg/mL] ± SD	
Sample	DPPH	H_2_O_2_
**As**	8.5 ± 1.9	7.8 ± 0.3
Myristicin	6.2 ± 1.2	7.1 ± 0.5
*β*-Phellandrene	14.8 ± 2.1	15.2 ± 0.9
Ascorbic acid	0.03 ± 0.03	0.04 ± 0.05

## Data Availability

The original data presented in the study are openly available in the original manuscript.

## Supplementary Materials

The following supporting information can be downloaded at https://www.mdpi.com/article/10.3390/antibiotics15010079/s1. Figure S1: ^1^H-NMR spectrum of myristicin (**1**); Figure S2: ^13^C-NMR spectrum of myristicin (**1**).
