# Supplementary material for: Myristicin from Athamanta sicula L.: A Potential Natural Antimicrobial Agent
Source: Antibiotics (Basel). 2026 Jan 13;15(1):79. doi: 10.3390/antibiotics15010079 (PMC12837313; doi:10.3390/antibiotics15010079)
Supplement: Supplementary file 1 [file antibiotics-15-00079-s001.zip › antibiotics-4086735-supplementary.pdf]

Supplementary Materials

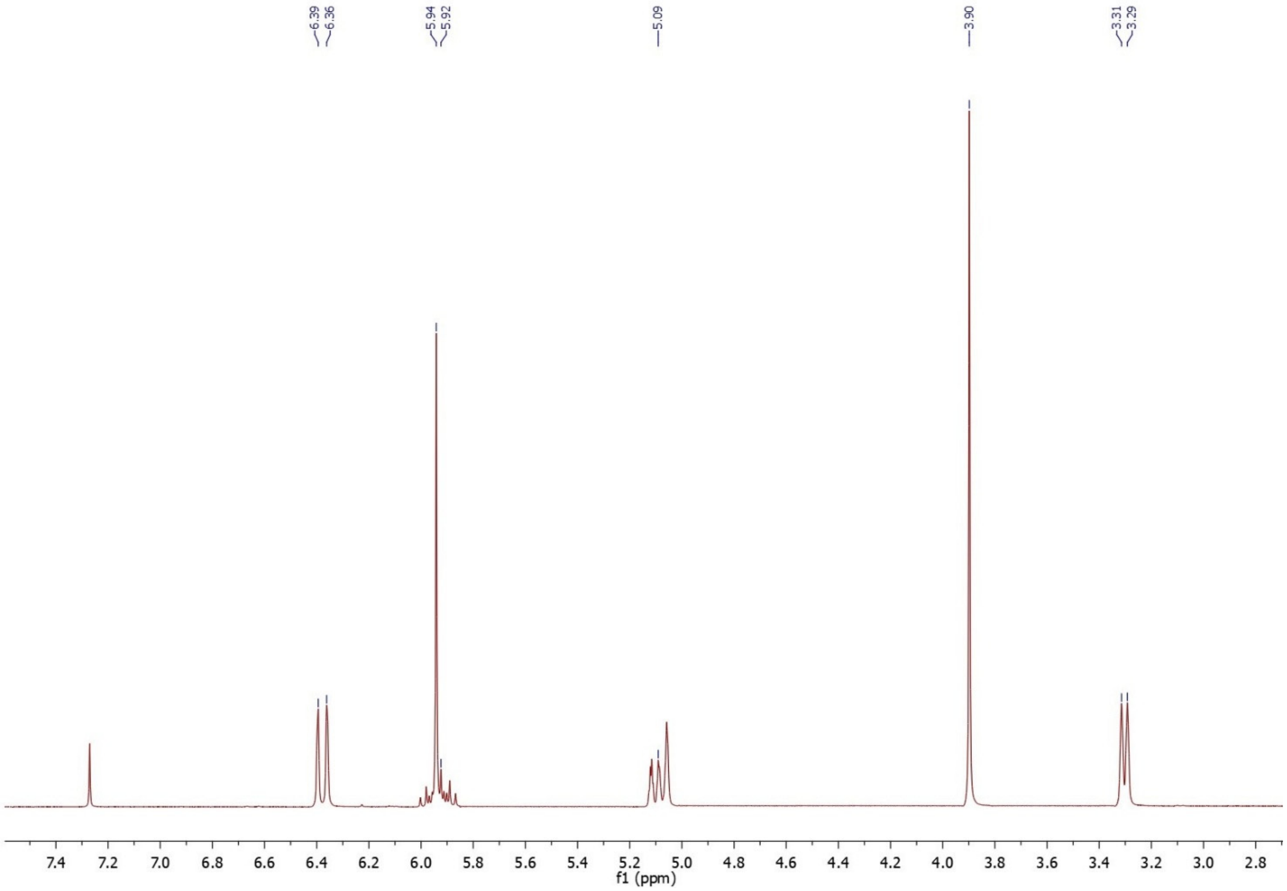

Figure S1. ^{1}H-NMR spectrum of myristicin (**1**).

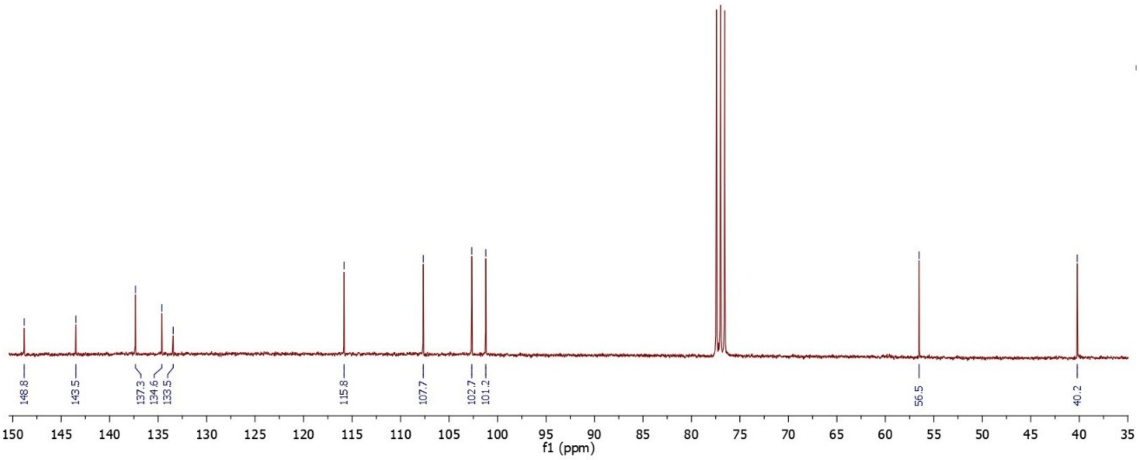

Figure S2. ^{13}C-NMR spectrum of myristicin (**1**).
